# Droplet digital PCR for detection and quantification of circulating tumor DNA in plasma of head and neck cancer patients

**DOI:** 10.1186/s12885-017-3424-0

**Published:** 2017-06-19

**Authors:** Joost H. van Ginkel, Manon M. H. Huibers, Robert J. J. van Es, Remco de Bree, Stefan M. Willems

**Affiliations:** 1 Department of Oral and Maxillofacial Surgery, University Medical Center Utrecht, Utrecht, The Netherlands; 2 Department of Pathology, University Medical Center Utrecht, Heidelberglaan 100, 3584 CX Utrecht, The Netherlands; 3 Department of Head and Neck Surgical Oncology, UMC Utrecht Cancer Center, University Medical Center Utrecht, Utrecht, The Netherlands

**Keywords:** Head and neck cancer, Circulating tumor DNA, Droplet digital PCR, *TP53* mutations, Diagnostic biomarker

## Abstract

**Background:**

During posttreatment surveillance of head and neck cancer patients, imaging is insufficiently accurate for the early detection of relapsing disease. Free circulating tumor DNA (ctDNA) may serve as a novel biomarker for monitoring tumor burden during posttreatment surveillance of these patients. In this exploratory study, we investigated whether low level ctDNA in plasma of head and neck cancer patients can be detected using Droplet Digital PCR (ddPCR).

**Methods:**

*TP53* mutations were determined in surgically resected primary tumor samples from six patients with high stage (II-IV), moderate to poorly differentiated head and neck squamous cell carcinoma (HNSCC). Subsequently, mutation specific ddPCR assays were designed. Pretreatment plasma samples from these patients were examined on the presence of ctDNA by ddPCR using the mutation-specific assays. The ddPCR results were evaluated alongside clinicopathological data.

**Results:**

In all cases, plasma samples were found positive for targeted *TP53* mutations in varying degrees (absolute quantification of 2.2–422 mutational copies/ml plasma). Mutations were detected in wild-type *TP53* background templates of 7667–156,667 copies/ml plasma, yielding fractional abundances of down to 0.01%.

**Conclusions:**

Our results show that detection of tumor specific *TP53* mutations in low level ctDNA from HNSCC patients using ddPCR is technically feasible and provide ground for future research on ctDNA quantification for the use of diagnostic biomarkers in the posttreatment surveillance of HNSCC patients.

**Electronic supplementary material:**

The online version of this article (doi:10.1186/s12885-017-3424-0) contains supplementary material, which is available to authorized users.

## Background

Monitoring tumor response during posttreatment surveillance of head and neck cancer patients heavily relies on clinical examination supported by endoscopy and/or imaging (e.g. computerized tomography (CT), magnetic resonance imaging (MRI), or positron emission tomography (PET)). However, early detection of recurrent disease is challenging due to lymph nodal micrometastases and radiation or surgery induced fibrosis and inflammation, obscuring residual or recurrent tumor tissue [1–3]. Accurate and timely detection of locoregional metastases and recurrent disease is pivotal as survival rates rapidly decline with late detection and delayed salvage surgery [4, 5]. With recent developments in molecular diagnostics, the use of (blood-based) genetic biomarkers is growing in a wide variety of cancer types [6]. Cell free circulating tumor DNA (ctDNA), released into the bloodstream by apoptotic and necrotic tumor cells, harbor tumor-specific mutations [7]. These mutations can be detected in blood plasma from cancer patients by blood sampling, also known as “liquid biopsy” [8]. For head and neck cancer, research has been focused mainly on actionable oncogenic mutations such as *PIK3CA* and *HRAS*, hot-spot *TP53* mutations, and HPV-related biomarkers to use as prognosticators or predictors for establishing and adjusting targeted therapy [9–12]. For similar purposes, transcriptional and epigenetic changes are studied substantially [13–15]. For the early detection of recurrent disease, early driver mutations in HNSCC such as TP53 mutations would be favorable to use as biomarkers, as these are likely to occur consistently throughout clonal evolution [16, 17], and are found to be most frequent and concordant in recurrent and metastatic HPV-negative tumors compared to mutations in other genes [18–22]. By targeting and quantifying early driver mutations in ctDNA, tumor burden could be monitored after treatment, facilitating earlier detection of asymptomatic residual and/or recurrent disease. Previous studies showed correlations between ctDNA levels and tumor dynamics during posttreatment monitoring in patients with various types of cancer [23–26]. However, accurate detection of ctDNA in plasma is challenging, because ctDNA concentrations can be very low. This could greatly impair reliable and valid measurement of tumor dynamics. Highly sensitive Droplet Digital PCR (ddPCR) facilitates detection and quantification of low levels of ctDNA by partitioning DNA samples into 20,000 water-in-oil droplets [27]. In this exploratory study, we investigated whether detection and quantification of ctDNA in plasma from several head and neck squamous cell carcinoma (HNSCC) patients using ddPCR is technically feasible.

## Methods

### Patients and samples

**Fig. 1:**
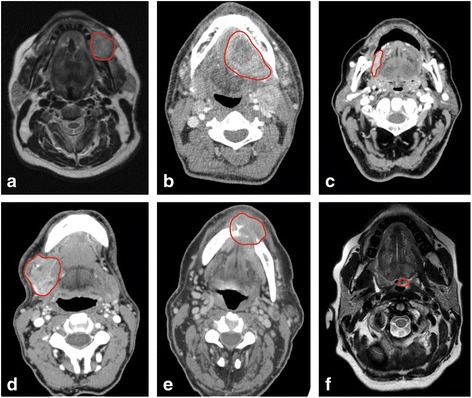
Primary tumors of six patients encircled in *red*. **a** Axial T1 MRI image of a tumor in the left mandible of patient 1. **b** Axial ceCT image of a tumor in the floor of mouth of patient 2. **c** Axial ceCT image of a tumor in the right lateral tongue of patient 3. **d** Axial ceCT image of a tumor in the right mandible/floor of mouth/tongue of patient 4. **e** Axial ceCT image of a tumor in the floor of mouth in patient 5. **f** Axial T1 MRI image of tumor in left mid tongue base of patient 6. ceCT = contrast enhanced computed tomography

Six patients (median age 60.5 [42–77] years) with histologically confirmed HPV-negative HNSCC were selected retrospectively for analysis of archived primary tumor samples and presurgically obtained blood samples. Patient selection was based on TNM stage (stage II or higher) and availability of blood plasma samples in our biobank. Additional clinicopathological and radiological data were collected from hospital charts of selected patients (Table 1; Fig. 1).

**Table 1:** Summary of patient and tumor characteristics

Patient ID	Sex	Smoking (pack years)	Alcohol (units/day)	Biopsy type	TNM-stage	Tumor site^a^	Differentiation grade	Max diameter primary tumor (mm)	Growth type^b^	Vascular invasion
P1	M	0	8	Excisional	T4aN1M0	OSCC	Moderate	40	NS	No
P2	M	0	0	Excisional	T4aN2cM0	OSCC	Poor	72	NS	Yes
P3	F	0	0	Excisional	T2N0Mx	OSCC	Moderate	32	Unknown	Yes
P4	M	Unknown	1	Excisional	T4aN2bM0	OSCC	Moderate	46	S	No
P5	M	49	12	Excisional	T4aN1M0	OSCC	Moderate/poor	37	Unknown	No
P6	F	42	2	Incisional	T3N2cM0	OPSCC	Unknown	13	N/A	No

^a^ *OSCC* Oral Squamous Cell Carcinoma, *OPSCC* Oropharyngeal Squamous Cell Carcinoma

^b^ *NS* Non Spiculated, *S* Spiculated

### Sample workup

All primary tumor samples were acquired from formalin fixed paraffin embedded (FFPE) incisional or excisional biopsy specimens, microscopically containing >30% tumor cells. In order to reveal *TP53* mutation status of primary tumor samples, targeted next-generation sequencing (NGS) was performed using the Ion Torrent™ PGM platform (Thermo Fisher Scientific, Waltham, MA, USA), as previously described [28]. NGS was based on the Cancer Hotspot Panel v2+ (Thermo Fisher Scientific, Waltham, MA, USA), covering *TP53* exons 2–10 [29]. All blood samples were collected in 10 ml K_2_EDTA blood collection tubes (BD Vacutainer, Franklin Lakes, NJ, USA). Prior to archiving, centrifugation took place for 10 min at 800 g (Rotina 380, Hettich, Germany), after which supernatant plasma was aliquoted in 1 ml portions and stored at −80 °C until DNA isolation. Storage time of patient FFPE and corresponding plasma samples varied from 4 months to 9 years.

Plasma samples were thawed and DNA was immediately isolated from 2 ml of plasma using QIAamp Circulating Nucleic Acid (NA) kit (Qiagen, Hilden, Germany) according to the manufacturer’s instructions. Isolated plasma samples were eluted in 50 μl elution buffer as provided with the kit and stored at 4 °C until ddPCR analysis. Positive control samples, containing both wild-type (WT) and mutant (MT) DNA, were created for all patients by isolating tumor DNA from the primary tumor FFPE samples using COBAS DNA Sample Preparation Kit (Roche, Basel, Switzerland) according to manufacturer’s instructions. After quantity measurement of isolated DNA samples with a Qubit fluorometer using the dsDNA HS (High Sensitivity) Assay Kit (Thermo Fisher Scientific), cfDNA was diluted to 10 ng/ul using purified water. For each assay, no template controls (NTC) were used to control for environmental contamination, and wild-type-only (WT-only) samples were used in order to estimate false-positive rates. Five WT-only samples were created by isolating plasma DNA from anonymous healthy individuals using the QIAamp Circulating NA kit.

### ddPCR

The plasma samples from all 6 patients were analyzed for *TP53* point mutations, identified in the primary tumor tissue by NGS. MT and WT *TP53* sequences were used as DNA template for designing ddPCR (Bio-Rad Laboratories, Hercules, CA, USA) assays following the MIQE guidelines (Additional file 1: Table S1) [30]. DdPCR reaction volumes of 22 μl were prepared, consisting of 13 μl mastermix (11 μl Supermix for Probes [no deoxyuridine triphosphate], 1 μl of primer/probe mix for both MT and WT *TP53*), and 9 μl cfDNA sample of patient plasma. The NTCs contained 9 μl of purified water instead of cfDNA sample. The WT-only samples contained 1–7 ul of cfDNA. From the PCR reaction mixture, 20 μl was used for droplet generation. Droplet Digital PCR was performed using the QX200 ddPCR system according to manufacturer’s instructions (Bio-Rad Laboratories). QuantaSoft v1.7.4.0917 (Bio-Rad Laboratories) software was used for data analysis.

Prior to plasma sample testing, thermal gradient experiments were performed on FFPE samples in order to determine optimal amplification conditions during thermal cycling for each assay independently. Based on clearest separation of negative and positive droplet clusters, thermal cycling conditions for all 6 assays were set at 95 °C for 10 min (1 cycle), 94 °C for 30 s and 55 °C for 60 s (55 cycles), and infinite hold at 12 °C. To ensure experiment quality, wells with total droplet counts of less than 10,000 would be considered invalid and excluded from analysis. The positive control samples were used to verify assay performance and facilitate thresholding in fluorescence values. Additionally, positive control samples were validated by comparing the fractional abundance (FA) in FFPE samples to NGS mutation frequencies. False-positive rate estimation was determined by performing 5 experiments for each assay using the WT-only samples, where total amounts of detected MT-positive droplets determined thresholds above which positive droplets in patient samples were to be considered as true positive.

### Post-analysis

For each patient, plasma was analyzed in duplicate. Therefore, PCR results of patients samples were based on the mean of estimated target DNA concentrations (copies/μl) in merged wells, automatically calculated by manufacturer software. Correction for false positivity was performed by virtually subtracting the amount of MT-false-positive droplets from the amount of MT-positive droplets detected in the patients sample with the corresponding assays. Subsequently, absolute sample concentrations were (re)calculated as described in Additional file 1: Eq. S1. Relative quantification was defined as the FA of MT to total (WT + MT) copies.

## Results

### Assay validation

In all six patients, *TP53* mutations were detected in FFPE by both NGS and ddPCR (Additional file 1: Table S1 and Additional file 2: Figure S1). FA of MT copies ranged from 6.1–71.7% in positive control samples, compared to NGS mutant percentages of 7–70%. False-positive rate estimation was necessary to determine aspecific MT signal (Additional file 1: Table S2). One MT-false-positive droplet was detected in the WT-only sample control series for assay 1 and 3, establishing a true positivity threshold of >1 MT-positive droplet for these assays (Additional file 3: Figure S2 and Additional file 4: Figure S3). For the remaining assays, no MT-false-positive droplets were detected in the WT-only samples. WT-false-positive droplets for all used assays in NTCs ranged from 0 to 10 droplets. No MT-positive droplets were detected in any of the NTC samples (Additional file 5: Figure S4).

### ctDNA quantification

**Fig. 2:**
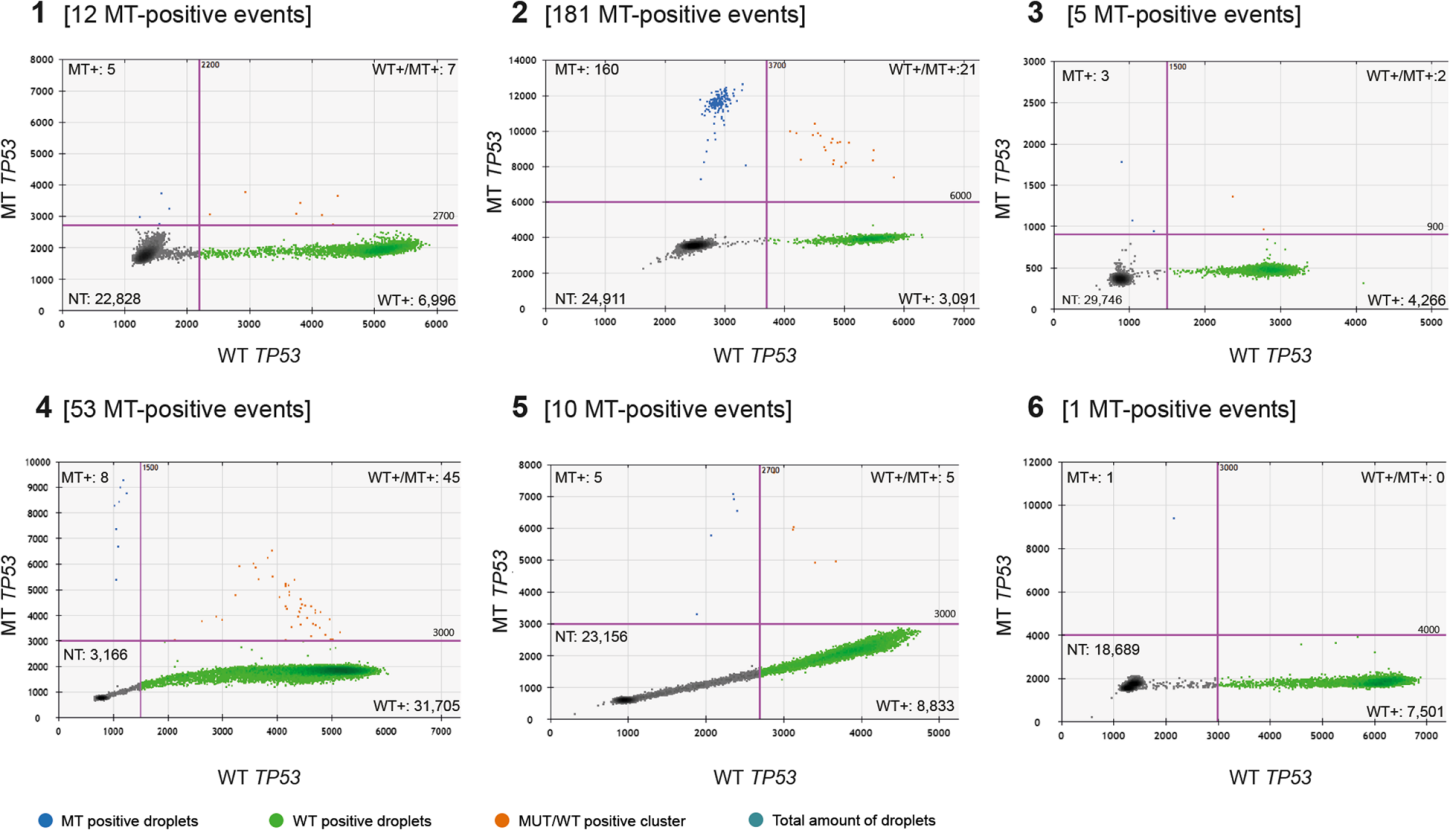
2D–plots and amount of MT-positive droplets of ddPCR results of all six patients. **a** All diagrams (1–6) represent merged ddPCR results of duplicates of corresponding patient samples (1–6), showing MT-positive droplet clusters (*blue dots*), negative droplet clusters (*dark grey dots*), and MT/WT-positive droplets (*orange dots*). The *green dots* represent WT-positive droplets, proving existence of cfDNA in the samples and satisfactory ddPCR conditions. *Purple lines* are manually placed thresholds for distinguishing positive and negative droplets, which were set at fluorescence values based on ddPCR results of FFPE samples. **b** The amount of MT-positive and negative droplets based on thresholds as placed in 2D–plots in (**a**)

**Fig. 3:**
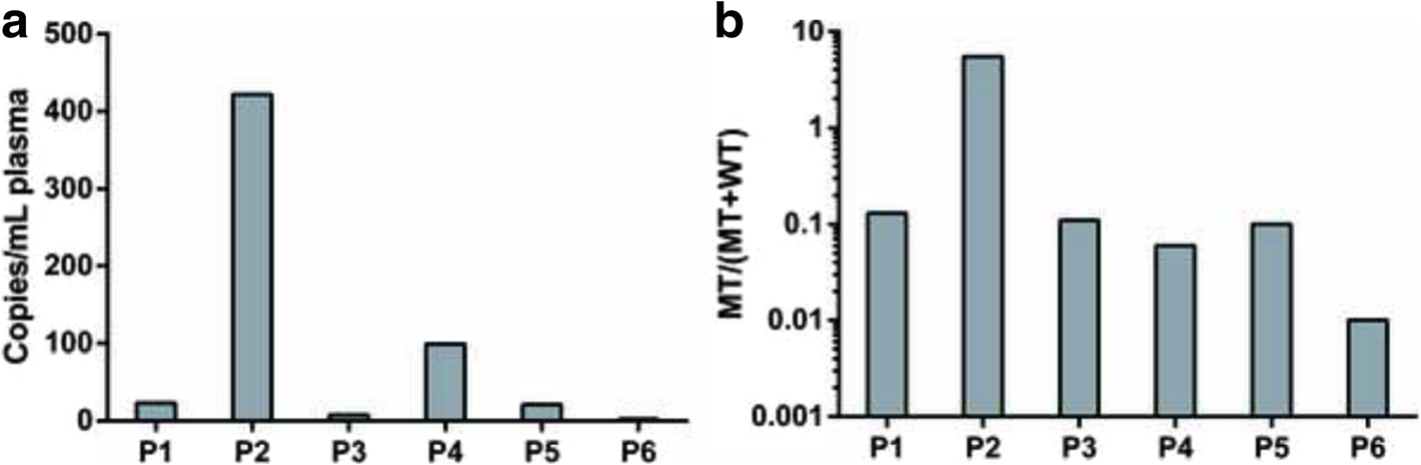
DdPCR results of patients (P1-P6) showing absolute quantification of ctDNA concentrations in plasma (**a**), and log-scaled fractional abundances of MT copies from total amount of MT and WT copies as corrected for total DNA input (**b**)

The amount of ctDNA was quantified and analyzed in blood plasma samples from all 6 patients (Table 2). MT copies of *TP53* were detected in plasma samples from all patients (Fig. 2a), ranging from 0.04 to 7.60 copies/μl ddPCR mix and 1–181 MT-positive droplets in merged wells (Fig. 2b). When corrected for MT-false-positive droplets, plasma ctDNA concentrations ranged from 2.2 to 422 copies/ml plasma (Fig. 3a). MT copies were detected in WT backgrounds of 138–2821 copies/μl, yielding FA of MT copies of 0.01–5.2% (Fig. 3b).

**Table 2:** Absolute and relative quantifications of MT and WT DNA in plasma samples from HNSCC patients

Sample ID	MT DNA concentration			WT DNA concentration		FA_mut_
	Sample (copies/μl)	Sample_corr_ (copies/μl)	Plasma (copies/ml)	Reaction (copies/μl)	Plasma (copies/ml)	
P1	0.47	0.43	24	315	17,500	0.13%
P2	7.60	7.60	422	138	7667	5.50%
P3	0.17	0.16	8.9	158	8778	0.10%
P4	1.79	1.79	99	2821	156,667	0.06%
P5	0.37	0.37	21	380	21,167	0.10%
P6	0.04	0.04	2.2	397	22,056	0.01%

## Discussion

Our study shows that quantification of rare target mutations in ctDNA in plasma from HNSCC patients using ddPCR is technically feasible. Highly sensitive detection methods like digital PCR are needed in order to detect rare MT targets within high concentrations of WT background [31]. WT background size (i.e. concentration of WT cfDNA) can strongly vary over time for each patient individually, depending on multiple factors. For instance, patient’s physical status (e.g. inflammation, post-traumatic, post-exercise, chronic illness), as well as pre-analytical technical procedures (e.g. white blood cell lysis caused by whole blood transportation and processing) appear to affect cfDNA concentrations [32–35]. Increased cfDNA concentration causes dilution of ctDNA, which could lower the accuracy of rare MT fragment detection. Therefore, pre-analytical steps should be most optimally in lowering background DNA; e.g. blood plasma instead of serum is preferred as source for ctDNA, as the amount of cfDNA in serum can be 2–4 times higher than that in plasma [36].

It has been shown for various applications that ddPCR is capable of rare target DNA quantification with higher precision and accuracy compared to quantitative PCR [27, 37–39]. Although we did not perform quantitative PCR we found relative quantification measurements of MT copies down to 0.01%. This falls within the potential dynamic range for absolute quantification of rare target DNA within a 100,000-fold of WT background as previously demonstrated [40, 41]. Similar quantification results were reported in a study where *TP53* mutations were identified in plasma using another PCR-based detection method in 88% of HPV-negative HNSCC patients (*n* = 22) with MT fractions varying between 0.016 and 2.9% [42]. We also found large variability in MT quantification measurements among patient samples. This is consistent with previous mutation analysis of blood samples from HNSCC patients, in which MT *TP53* fragments of 0–1500 per 5 ml plasma were targeted and detected by conventional PCR [43].

Variances in detected MT copies among patients can be the result of various (pre)analytical deficiencies and technical errors like plasma sample contamination from the environment. Furthermore, decreased DNA concentration due to prolonged storage, poor sample quality, subsampling during whole blood retrieval and/or centrifugation, inefficient DNA isolation from plasma samples, poor droplet handling leading to shredding or coalition of droplets, instrument artifacts, intrinsic PCR errors caused by PCR inhibition and/or minor mismatches between primer/probes and target molecules can all affect PCR results [44, 45].

During ddPCR post-analysis, manual threshold determination and stochastic sampling errors could directly lead to over- or underestimation of target copies, resulting in inaccurate quantification of results [46]. Furthermore, we know from previous validation experiences that fluorescence values of positive droplet clusters can vary inter-experiment, while assessing DNA samples derived from the same individual and using identical ddPCR assays. The same holds true for ddPCR experiments on DNA samples derived from different plasma matrices and/or volumes, containing different PCR inhibitors [47]. These points concerning post-analysis need to be addressed in order to implement ddPCR for ctDNA quantification into clinical practice. Therefore each assay and each sample should be analyzed individually. Although we used FFPE for positive control samples for threshold placement and plasma from different individuals for false-positive rate estimation, samples were patient specific and of similar matrix of DNA source, respectively. In this way, plasma DNA composition from the patients was mimicked most realistically. Moreover, the alternative of using (spiked) series of artificially synthesized DNA oligonucleotides for creating control samples can provoke overestimation of PCR targets due to the high purity of these solutions. Eventually, interpretation of ddPCR results depends on the accuracy of ctDNA quantification which is determined by false positive rate estimation.

Several biological factors could affect ctDNA concentration. Especially tumor volume is of interest as it may reflect tumor burden and actual disease status through correlation with ctDNA concentration. Simultaneously, tumor characteristics such as histological grade, localization, growth pattern, growth rate, and degree of vascularization possibly complicate reliable monitoring of tumor burden by ctDNA quantification, as these factors might affect ctDNA release into the bloodstream all differently [44, 48]. However, in a series of 117 patients with primary HNSCC, no significant correlation was found between gender, tumor stage, site, and plasma ctDNA concentration detected by touchdown PCR [49]. Interestingly, in our study, the highest amount of ctDNA was detected in plasma from the patient that harbored the largest tumor diameter of all six included patients. This tumor also had a poor histological differentiation grade with vascular invasion. At the other end, the lowest amount of ctDNA was detected in plasma from the patient with the smallest tumor diameter and without vascular invasion. However, we studied and compared plasma samples retrieved at one time point from a rather small group of high-stage HNSCC patients with presumably greater tumor burden and plasma ctDNA concentrations.

Therefore, serial ctDNA quantification in clinical patients diagnosed with primary HNSCC of all stages is needed to clarify its significance for posttreatment disease monitoring and the possible advantages of its specific application with respect to early tumor detection in relation to current clinical diagnostics [50]. Tumor heterogeneity could further complicate monitoring tumor burden through ctDNA detection, because intratumoral heterogeneity of the primary tumor induces branched tumor evolution of subclonal populations harboring different molecular alterations [51]. This could lead to increased clonal heterogeneity between primary tumor and matched metastatic or recurrent tumors, risking mistargeting of ctDNA. However, as early driver *TP53* mutations show high concordance between primary and recurrent and/or metastatic tumors, these may hold promise as most reliable targets for ctDNA detection and for early tumor detection of HNSCC recurrences [21].

## Conclusion

The detection of tumor specific *TP53* mutations in ctDNA from HNSCC using a ddPCR is technically feasible and provide ground for further research on ctDNA quantification to be used as a diagnostic biomarker in the posttreatment surveillance of HNSCC patients.

## Additional files

Additional file 1: Table S1–2. NGS data, PCR assays, and Assay validation. Eq. S1 Equation used for manual conversion of target copies to plasma concentrations. (DOCX 24 kb)

Additional file 2: Figure S1. DdPCR results of 6 different MT *TP53* assays on positive control (FFPE) samples of all 6 patients are shown. The MT-positive clusters (blue dots) and MT/WT-positive clusters (orange dots) are clearly separated from the negative droplet clusters (dark grey dots) and WT-positive droplet clusters. Thresholds are placed manually. (TIFF 1834 kb)

Additional file 3: Figure S2. 2D–plots with the amounts of droplets of ddPCR results in healthy individuals using assay 1–6. All threshold are placed using exact values as derived from the 2D–plots in Additional file 2: Figure S1. The plots represent merged results of plasma samples from 4 to 5 different healthy individuals for each assay. MT+ MT-positive droplets, WT+ WT-positive droplets, MT+/WT+ MT/WT-positive droplets, NT No template droplets. (TIFF 1255 kb)

Additional file 4: Figure S3. DdPCR results for all 6 patients side-by-side with the WT-only samples from healthy individuals. All patient samples are shown in duplicate. In order to estimate the false positive rate for patient samples, plasma samples from five different healthy individuals were used. In the samples from healthy individuals 3 and 1 used during validation of assay 2 and assay 6, less than 10,000 droplets were detected. Therefore, these results were excluded from false positive estimation for the corresponding assays. (TIFF 6899 kb)

Additional file 5: Figure S4. NTC samples showing minimal environmental contamination with WT-positive droplets. No MT-positive droplets were detected in any of the NTC samples. (TIFF 3242 kb)

## Abbreviations

CT: Computer tomography
ctDNA: Circulating tumor DNA
ddPCR: Droplet digital polymerase chain reaction
FA: Fractional abundance
FFPE: Formalin fixed paraffin embedded
HNSCC: Head and neck squamous cell carcinoma
HPV: Human papilloma virus
MRI: Magnetic resonance imaging
MT: Mutant
NGS: Next-generation sequencing
PET: Positron emission tomography
WT: Wild type
