# Supplementary material for: Droplet digital PCR for detection and quantification of circulating tumor DNA in plasma of head and neck cancer patients
Source: BMC Cancer. 2017 Jun 19;17:428. doi: 10.1186/s12885-017-3424-0 (PMC5477260; doi:10.1186/s12885-017-3424-0)
Supplement: Supplementary file 1 — NGS data, PCR assays, and Assay validation. Eq. S1 Equation used for manual conversion of target copies to plasma concentrations. (DOCX 24 kb) [file 12885_2017_3424_MOESM1_ESM.docx]

**Table S1.** NGS data and PCR assays

| Patient ID | Gene | Mutation | Mutation effect | Mutant protein | NGS mutation  frequency | ddPCR  Mutation FA | PCR assay | MIQE Context [wt/mut] | Amplicon length |
| --- | --- | --- | --- | --- | --- | --- | --- | --- | --- |
| 1 | *TP53* | c.920-1G>T | Splice acceptor | Unknown | 19% | 22.4% | COSM95740 | AGACCAAGGGTGCAGTTATGCCTCAGATTCACTTTTATCACCTTTCCTTGCCTCTTTCCTA[G/T]CACTGCCCAACAACACCAGCTCCTCTCCCCAGCCAAAGAAGAAACCACTGGATGGAGAATA | 68 bp |
| 2 | *TP53* | c.700T>G | Missense | p.T234A | 44% | 34.3% | COSM146344 | ACTGGCCTCATCTTGGGCCTGTGTTATCTCCTAGGTTGGCTCTGACTGTACCACCATCCAC[T/G]ACAACTACATGTGTAACAGTTCCTGCATGGGCGGCATGAACCGGAGGCCCATCCTCACCAT | 64 bp |
| 3 | *TP53* | c.526T>C | Missense | p.C176A | 7% | 6.1% | COSM44948 | CCGCGTCCGCGCCATGGCCATCTACAAGCAGTCACAGCACATGACGGAGGTTGTGAGGCGC[T/C]GCCCCCACCATGAGCGCTGCTCAGATAGCGATGGTGAGCAGCTGGGGCTGGAGAGACGACA | 65 bp |
| 4 | *TP53* | c.625A>T | Stop gained | p.I255P | 20% | 42.5% | COSM43651 | CAACTACATGTGTAACAGTTCCTGCATGGGCGGCATGAACCGGAGGCCCATCCTCACCATC[A/T]TCACACTGGAAGACTCCAGGTCAGGAGCCACTTGCCACCCTGCACACTGGCCTGCTGTGCC | 64 bp |
| 5 | *TP53* | c.763A>T | Missense | p.A209* | 50% | 46.6% | COSM11290 | GGCCCCTCCTCAGCATCTTATCCGAGTGGAAGGAAATTTGCGTGTGGAGTATTTGGATGAC[A/T]GAAACACTTTTCGACATAGTGTGGTGGTGCCCTATGAGCCGCCTGAGGTCTGGTTTGCAAC | 65 bp |
| 6 | *TP53* | c.1015G>T | Missense | p.E339* | 70% | 71.7% | COSM214290 | TGTGTATATACTTACTTCTCCCCCTCCTCTGTTGCTGCAGATCCGTGGGCGTGAGCGCTTC[G/T]AGATGTTCCGAGAGCTGAATGAGGCCTTGGAACTCAAGGATGCCCAGGCTGGGAAGGAGCC | 65 bp |

**Equation S1.** Absolute quantification was determined by calculating the number of copies of target DNA per ml plasma using the sample concentrations:

$$C=-\ln\left( \frac{N_{neg}}{N} \right)/V_{droplet}$$

*C* = sample concentration (copies/µl)

*N_neg_* = number of negative droplets

*N* = total number of droplets

*V_droplet_* = volume of droplet (0.85 nl)

$$PC=C \cdot RV \cdot\frac{EV}{TV}/PV$$

*PC* = plasma concentration (copies/ml)

*C* = sample concentration (copies/µl)

*RV* = PCR reaction volume (20 µl)

*EV* = volume in which cfDNA was eluted (50 µl);

*TV* = volume of cfDNA added to the PCR reaction (9 µl)

PV = volume of plasma used for cfDNA extraction (2 ml)

**Table S2.** Assay validation: limit of detection (LOD)

|  | Sample | Assay 1 | Assay 2 | Assay 3 | Assay 4 | Assay 5 | Assay 6 |
| --- | --- | --- | --- | --- | --- | --- | --- |
| Fluorescence amplitude (MUT/WT) |  | 2700/2200 | 6000/3700 | 900/1500 | 3000/1500 | 3000/2700 | 4000/3000 |
| # Positive droplets in WT-only samples (MUT/WT) | 1 | 0/2794 | 0/2762 | 1/3632 | 0/3138 | 0/3212 | N/A |
|  | 2 | 1/12892 | 0/16515 | 0/15167 | 0/17775 | 0/16314 | 0/18230 |
|  | 3 | 3/836 | N/A | 0/921 | 0/834 | 0/1035 | 0/814 |
|  | 4 | 0/943 | 0/656 | 0/1072 | 0/855 | 0/822 | 0/733 |
|  | 5 | 0/518 | 0/555 | 0/794 | 0/651 | 0/580 | 0/696 |
| MT-false-positive droplets |  | 4 | 0 | 1 | 0 | 0 | 0 |
| Mean false-positive concentration (copies/µl) |  | 0.064 | 0 | 0.012 | 0 | 0 | 0 |
| # Positive droplets in NTC samples (MUT/WT) | 1 | 0/1 | 0/4 | 0/1 | 0/3 | 0/0 | 0/0 |
|  | 2 | 0/0 | N/A | 0/0 | 0/2 | 0/0 | 0/0 |
|  | 3 | 0/1 | 0/2 | 0/1 | 0/4 | 0/0 | 0/0 |
|  | 4 | 0/2 | 0/0 | 0/5 | 0/1 | 0/0 | 0/1 |
| Mean positive droplets |  | 0/1 | 0/2 | 0/2 | 0/3 | 0/0 | 0/0 |

N/A = not analyzed (total droplet count < 10,000)
