# Supplementary figures and images for: Droplet digital PCR for detection and quantification of circulating tumor DNA in plasma of head and neck cancer patients
Source: BMC Cancer. 2017 Jun 19;17:428. doi: 10.1186/s12885-017-3424-0 (PMC5477260; doi:10.1186/s12885-017-3424-0)

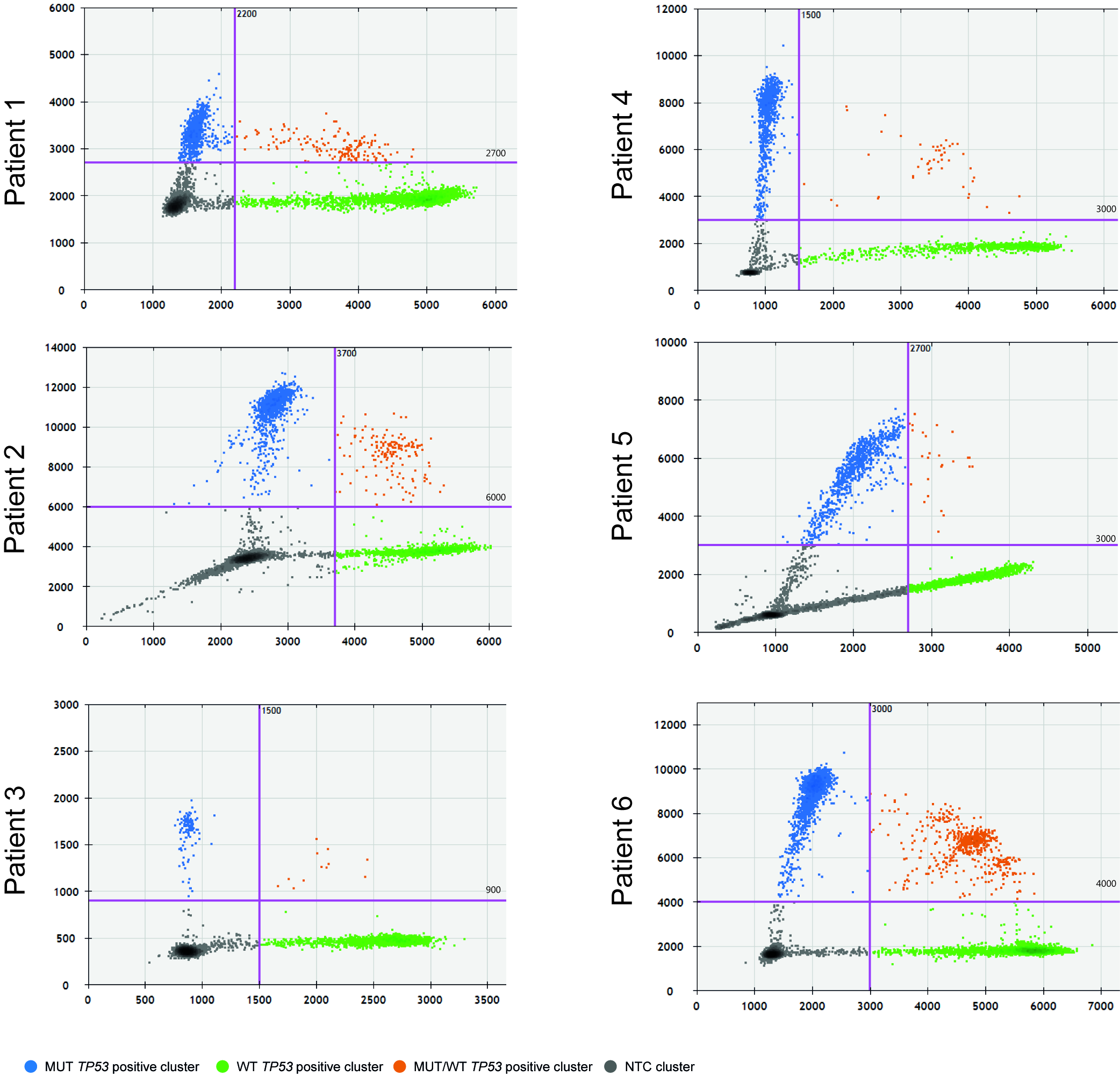

Supplement: Supplementary file 2 — DdPCR results of 6 different MT TP53 assays on positive control (FFPE) samples of all 6 patients are shown. The MT-positive clusters (blue dots) and MT/WT-positive clusters (orange dots) are clearly separated from the negative droplet clusters (dark grey dots) and WT-positive droplet clusters. Thresholds are placed manually. (TIFF 1834 kb) [file 12885_2017_3424_MOESM2_ESM.tif]

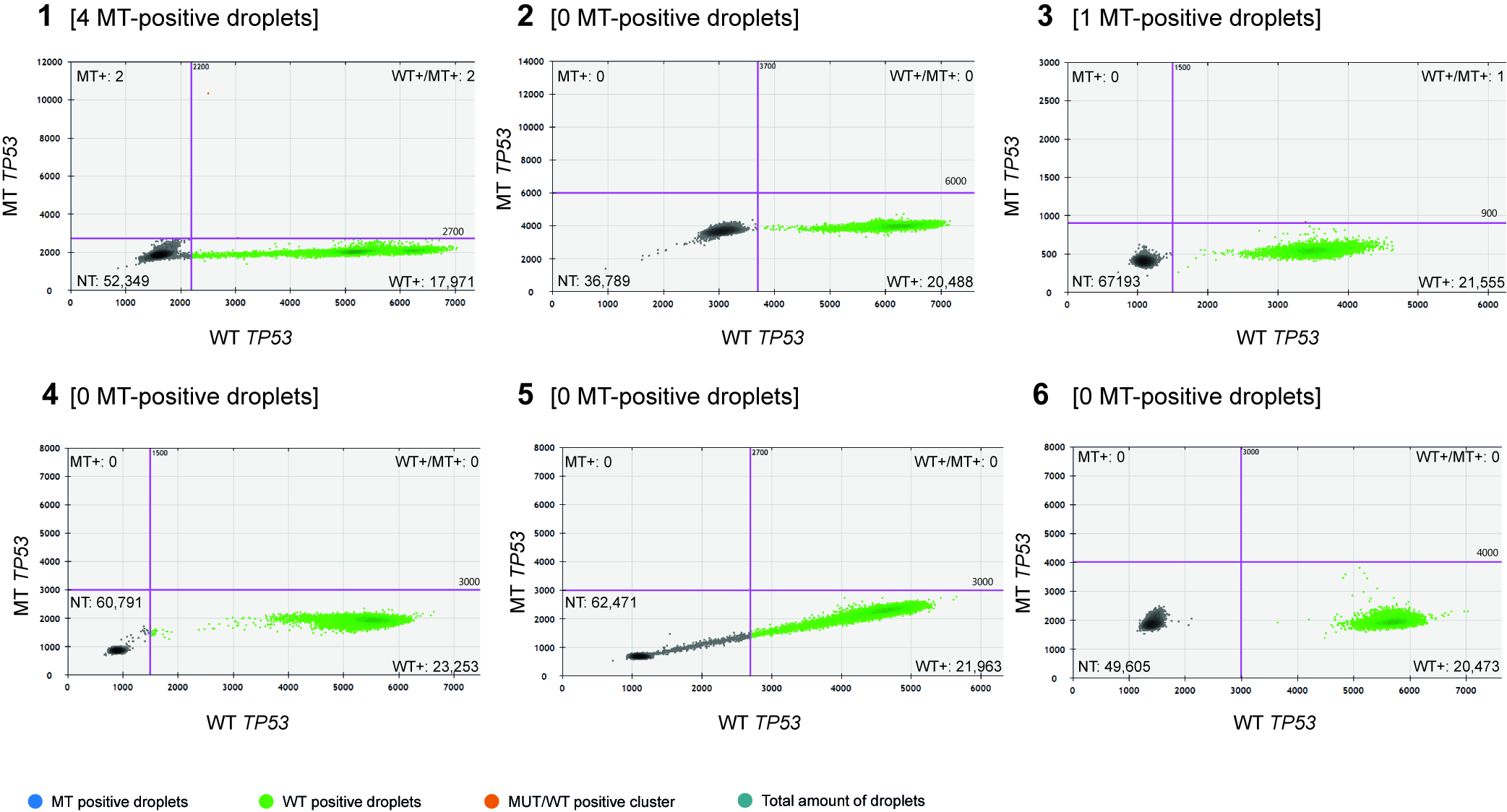

Supplement: Supplementary file 3 — 2D–plots with the amounts of droplets of ddPCR results in healthy individuals using assay 1–6. All threshold are placed using exact values as derived from the 2D–plots in Additional file 2: Figure S1. The plots represent merged results of plasma samples from 4 to 5 different healthy individuals for each assay. MT+ MT-positive droplets, WT+ WT-positive droplets, MT+/WT+ MT/WT-positive droplets, NT No template droplets. (TIFF 1255 kb) [file 12885_2017_3424_MOESM3_ESM.tif]

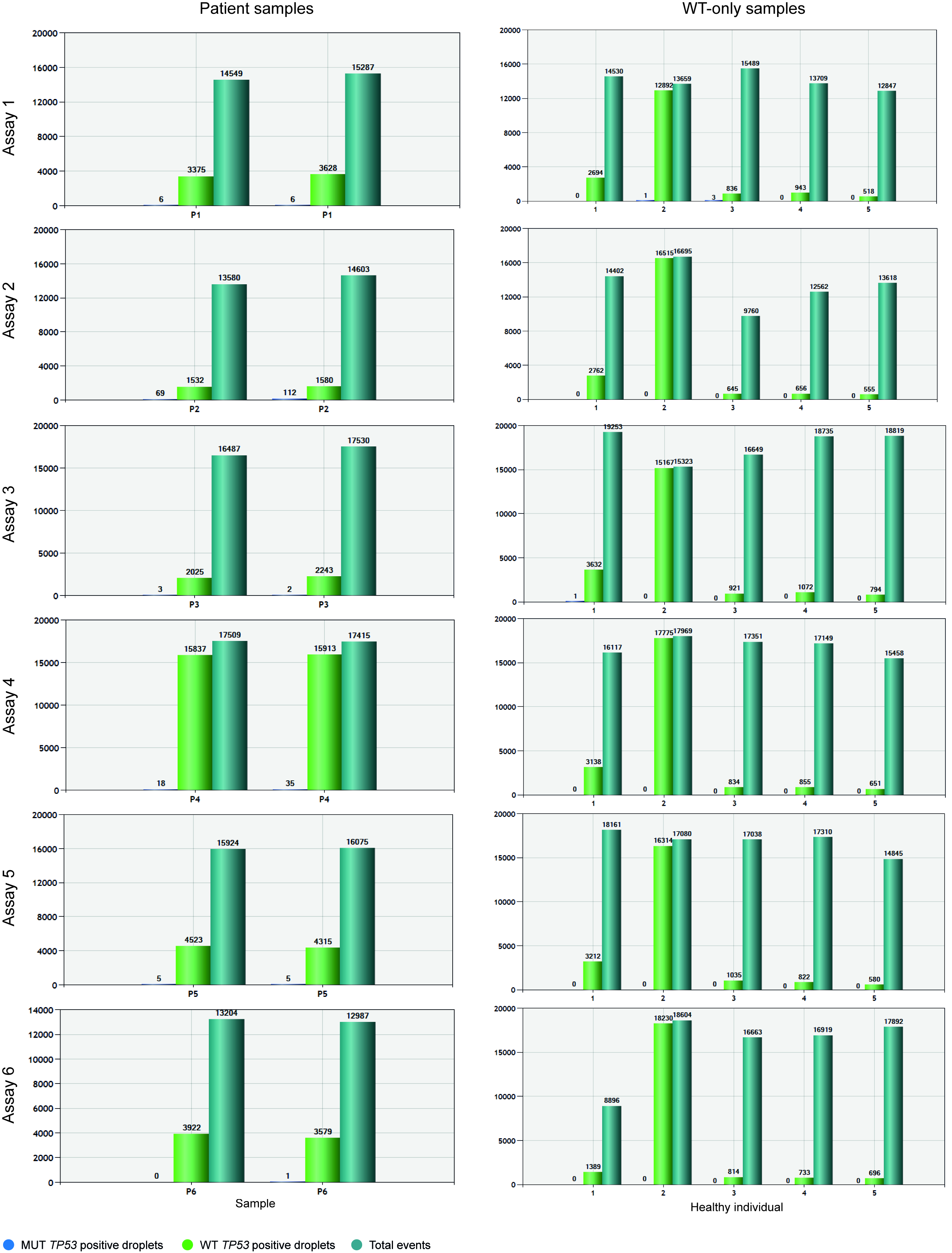

Supplement: Supplementary file 4 — DdPCR results for all 6 patients side-by-side with the WT-only samples from healthy individuals. All patient samples are shown in duplicate. In order to estimate the false positive rate for patient samples, plasma samples from five different healthy individuals were used. In the samples from healthy individuals 3 and 1 used during validation of assay 2 and assay 6, less than 10,000 droplets were detected. Therefore, these results were excluded from false positive estimation for the corresponding assays. (TIFF 6899 kb) [file 12885_2017_3424_MOESM4_ESM.tif]

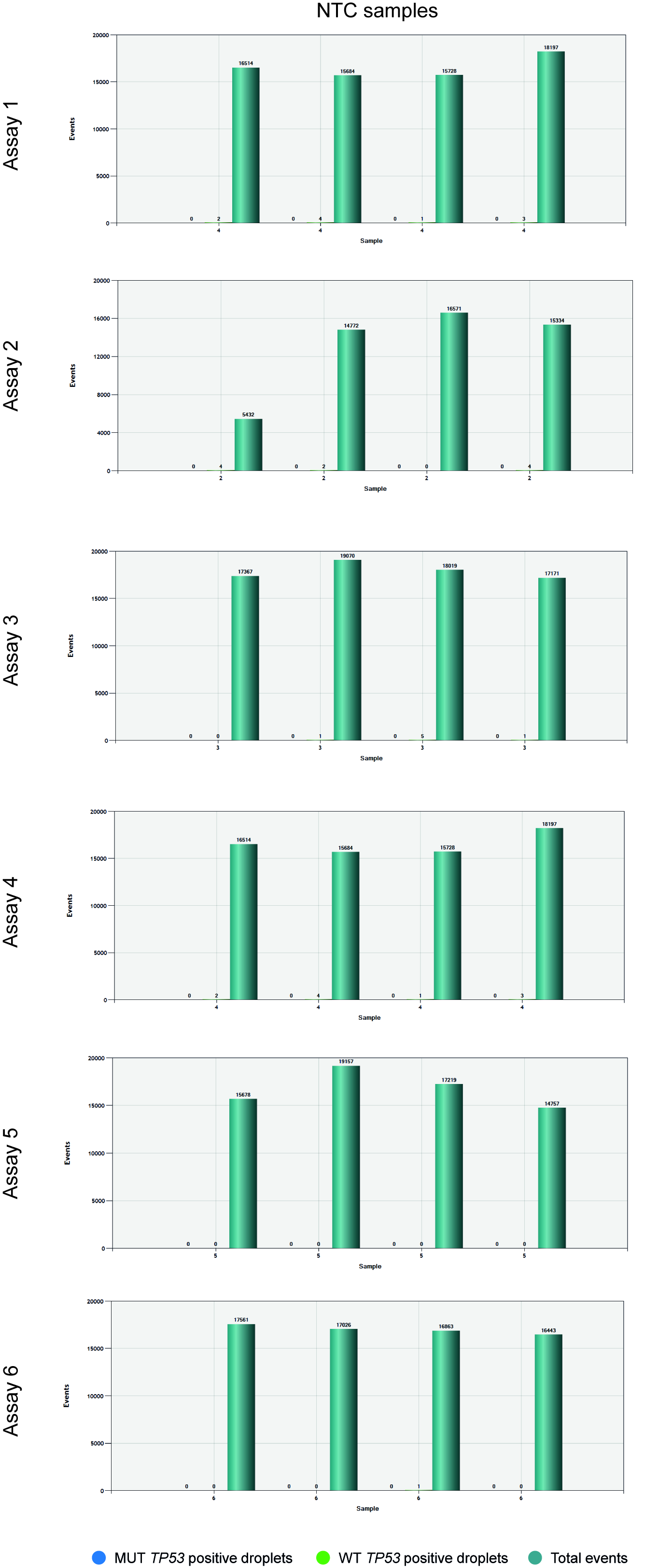

Supplement: Supplementary file 5 — NTC samples showing minimal environmental contamination with WT-positive droplets. No MT-positive droplets were detected in any of the NTC samples. (TIFF 3242 kb) [file 12885_2017_3424_MOESM5_ESM.tif]
